# ETAS^®^, a Standardized Extract of *Asparagus officinalis* Stem, Alleviates Sarcopenia via Regulating Protein Turnover and Mitochondrial Quality

**DOI:** 10.3390/ph18091243

**Published:** 2025-08-22

**Authors:** Sue-Joan Chang, Yung-Chia Chen, Yun-Ching Chang, Chung-Che Cheng, Yin-Ching Chan

**Affiliations:** 1 Department of Life Sciences, College of Bioscience and Biotechnology, National Cheng Kung University, Tainan 70101, Taiwan; 2 Marine Biology and Cetacean Research Center, National Cheng Kung University, Tainan 70101, Taiwan; 3 Department of Anatomy, School of Medicine, College of Medicine, Kaohsiung Medical University, Kaohsiung 80708, Taiwan; yungchia@kmu.edu.tw; 4 Graduate Institute of Medicine, College of Medicine, Kaohsiung Medical University, Kaohsiung 80708, Taiwan; 5 Department of Medical Research, Kaohsiung Medical University Hospital, Kaohsiung 80708, Taiwan; 6 School of Medicine, College of Medicine, I-Shou University, Kaohsiung 82445, Taiwan; ychang014@isu.edu.tw; 7 Department of Food and Nutrition, Providence University, Taichung 43330, Taiwan; ycchan@pu.edu.tw

**Keywords:** *Asparagus officinalis*, ETAS^®^, aging, sarcopenia, autophagy, mitochondrial quality

## Abstract

**Background**: ETAS^®^, a standardized extract of *Asparagus officinalis* stem, has been found to alleviate cognitive impairment in senescence-accelerated mice prone 8 (SAMP8) and is now considered a functional food in aging. The present study aimed to investigate the impacts of ETAS^®^ on relieving aging-related muscle atrophy in SAMP8 mice. **Methods**: The SAMP8 mice were fed a regular diet supplemented with 200 or 1000 mg/kg BW ETAS^®^50 for 12 weeks. Grip strength, muscle mass, and molecular markers of protein synthesis, degradation, and mitochondrial quality were assessed. **Results**: We found that ETAS^®^ significantly increased grip strength and muscle mass in SAMP8 mice. At the molecular level, ETAS^®^ significantly upregulated protein synthesis via PI3K/Akt/mTOR/p70S6K and downregulated protein degradation via FoxO1a/atrogin-1 and MuRF-1 and myostatin via NFκB expression. In addition, ETAS^®^ improved mitochondrial quality via promoting mitochondrial biogenesis genes, oxidative respiration genes, fusion/fission genes, PGC1α, and PINK1 proteins and maintained the autophagic flux via reducing ATG13, LC3-II/LC3-I, and p62. **Conclusions**: ETAS^®^ exerts beneficial effects on sarcopenia by modulating the positive protein turnover and improving mitochondrial quality in aging.

## 1. Introduction

*Asparagus officinalis,* commonly known as asparagus, is a perennial flowering plant belonging to the *Asparagaceae* family and is widely cultivated for its young shoots, a nutrient-rich vegetable [1]. A standardized extract of *A. officinalis* stems, trademarked as ETAS^®^ (Amino Up Co., Ltd., Sapporo, Hokkaido, Japan), has been recommended as a functional food due to its pharmacological activities, such as antioxidant [2], anti-inflammatory [3], neuroprotective [4,5], and sleep-enhancing properties [6]. ETAS^®^ safety evaluations have been conducted [7,8], and no severe effects have been observed in healthy individuals [8]. Our previous study provides evidence supporting the use of ETAS^®^ to reduce cognitive impairment progression, decrease amyloid-beta deposition, and enhance circadian rhythm signaling in aging. ETAS^®^ exerts anti-inflammatory effects by inhibiting nuclear factor kappa-B (NFκB) [9,10] and pro-inflammatory cytokines [3,10,11] and displays antioxidant properties by upregulating heat shock protein 70 (HSP70) [2,6,12] and maintaining redox balance [2,12]. Additionally, it mitigates oxidative and inflammatory damage, highlighting its therapeutic promise for aging-associated diseases.

Skeletal muscle is essential for movement, posture maintenance, and overall physical function [13]. It is pivotal in supporting daily activities and in contributing to a healthy and active lifestyle [13]. Aging results in a gradual decline in skeletal muscle mass, strength, and function, a condition referred to as sarcopenia [14,15]. Sarcopenia is marked by involuntary muscle loss, decreased strength, and impaired physical performance, with low muscle strength being a primary diagnostic criterion [15].

Aging-related sarcopenia is a multifactorial process driven by impaired protein synthesis, increased protein degradation, mitochondrial dysfunction, oxidative stress, and chronic inflammation [16]. With age, skeletal muscles become less responsive to anabolic signals, leading to a decreased activation of the Phosphoinositide 3-kinase (PI3K)/Akt and mammalian target of rapamycin (mTOR) pathways, further suppressing protein synthesis [16]. Simultaneously, the ubiquitin-proteasome system (UPS), which is responsible for protein degradation, is notably increased in aging muscle [17]. During sarcopenia, E3 ubiquitin ligases, such as Muscle RING-finger protein-1 (MuRF-1) and muscle atrophy F-box (MAFbx, also known as atrogin-1), are upregulated and they target myofibrillar proteins for degradation, leading to muscle wasting [17]. Additionally, the forkhead box O (FoxO) transcription factors (FoxO1a and FoxO3a), which regulate the expression of MuRF-1 and atrogin-1, become more active with aging, thereby accelerating protein degradation [17].

In addition to the UPS, autophagy plays a crucial role in the degradation of proteins [18,19]. In aged skeletal muscle, the autophagic function becomes impaired. It cannot effectively clear dysfunctional proteins and organelles, particularly mitochondria, which are vital for maintaining the integrity of skeletal muscle fibers [20]. Impaired autophagy accumulates dysfunctional mitochondria and misfolded proteins, exacerbating oxidative stress and muscle atrophy [20]. Dysregulation of mitochondrial autophagy (mitophagy) deteriorates muscle atrophy in aging-related sarcopenia [20].

Mitochondrial dysfunction is now recognized as a critical factor in the progression and worsening of sarcopenia. [20]. During aging, mitochondrial biogenesis, fusion/fission dynamics, and mitophagy (selective mitochondrial autophagy) are impaired, leading to the accumulation of dysfunctional mitochondria and increased oxidative stress [21]. Our previous study found that SAMP8 mice exhibited typical features of sarcopenia at 40 weeks of age, indicating decreased muscle mass and strength. In addition, disturbance of mitochondrial quality control and exacerbation of autophagic flux at an early age can lead to the progression of sarcopenia [22].

The effect of ETAS^®^ on alleviating aging-induced sarcopenia remains unclear. The present study aimed to investigate how ETAS^®^ alleviates sarcopenia in senescence-accelerated mouse prone 8 (SAMP8) and the associated mechanisms.

## 2. Results

### 2.1. ETAS^®^ Enhances Grip Strength and Muscle Mass in SAMP8 Mice

Skeletal muscle strength and mass decline with advancing age [23]. Our group found that SAMP8 exhibited features of sarcopenia with decreased muscle strength and mass, indicating that SAMP8 is a reliable and time-saving animal model for studying sarcopenia [22].

**Figure 1:**
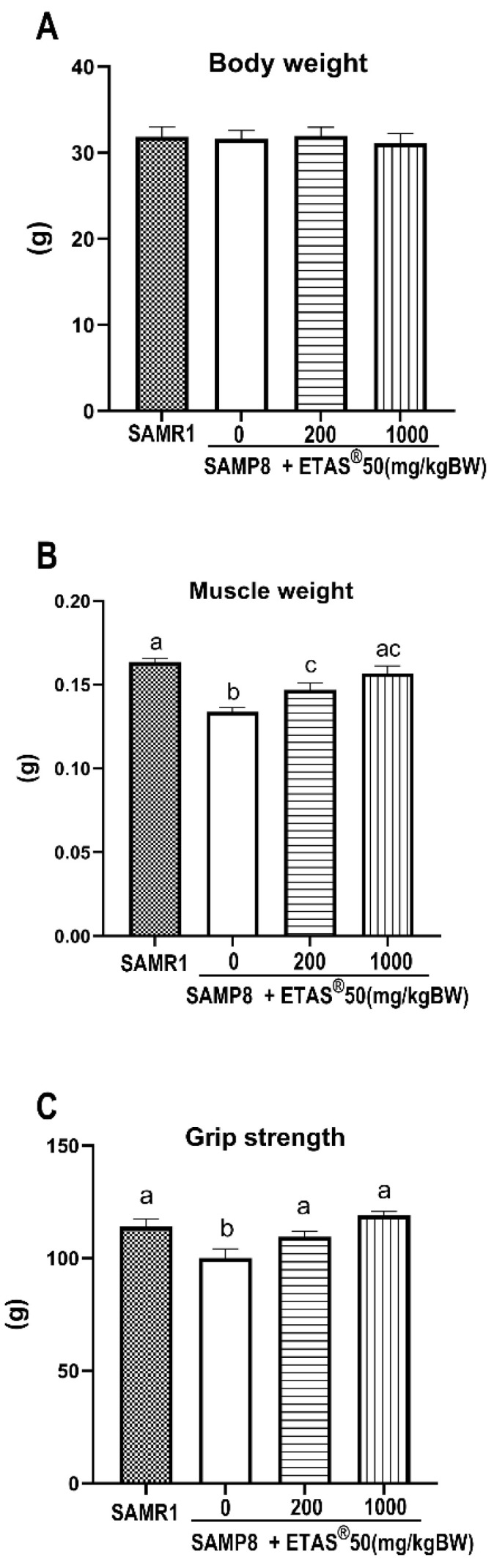
(**A**) Body weight, (**B**) muscle mass, and (**C**) grip strength of SAMP8 mice. Results are presented as means ± SEM for eight mice per group. Significance (*p* < 0.05) among groups is indicated by different letters.

The dosage of ETAS^®^ used in this study was determined based on the findings of Ito et al. (2014) [12], which showed that ETAS^®^ helped alleviate stress caused by sleep deprivation in mice. The chosen dose fell within the effective range reported in that study and was deemed suitable for assessing the physiological effects of ETAS^®^ in the context of aging-related muscle atrophy. Body weight (Figure 1A) was not changed among the groups. SAMP8 mice fed a standard chow diet for 12 weeks showed significantly reduced gastrocnemius muscle mass and grip strength compared to the control SAMR1 mice (Figure 1B,C). After 12 weeks of ETAS^®^50 (200 and 1000 mg/kg BW) supplementation, the grip strength and gastrocnemius muscle mass of SAMP8 mice (24-week-old) exhibited a noticeable improvement over that of SAMR1 (Figure 1B,C).

### 2.2. ETAS® Promotes Protein Synthesis via PI3K/Akt/mTOR/p70S6K in Skeletal Muscle of SAMP8 Mice

**Figure 2:**
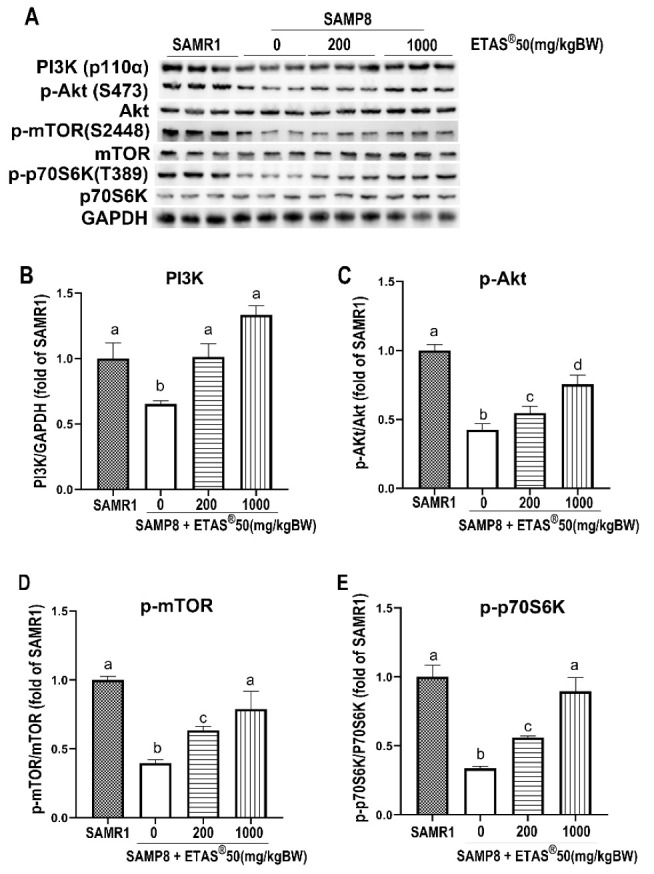
The expression of proteins associated with muscle protein synthesis was assessed using Western blot analysis. (**A**) displays representative images, while (**B**–**E**) provide quantification of the proteins PI3K, pAkt, pmTOR, and pp70S6K. Results are presented as means ± standard error of the mean (SEM). Each experimental group consisted of 4 to 6 mouse samples, with GAPDH utilized as a loading control. Differences in statistical significance (*p* < 0.05) among the groups are indicated by different letters.

Supplementation of ETAS^®^ enhanced skeletal muscle strength and gastrocnemius muscle mass in SAMP8 mice (Figure 1B,C). We further assessed the anabolic signaling pathways involved in muscle maintenance and found that phospho-PI3K, -Akt, -mTOR, and -p70S6K were significantly lower in non-treated SAMP8 mice than age-matched SAMR1 mice (Figure 2A–E), reflecting impaired anabolic signaling in aging. Translation of mRNA in mammalian cells is a significant step in protein synthesis, and mTOR controls the convergent point of it [24]. ETAS^®^50 (200 and 1000 mg/kg BW) supplementation for 12 weeks significantly elevated phospho-PI3K, -Akt, -mTOR, and -p70S6K in SAMP8 mice compared to non-treated SAMP8 mice (Figure 2A–E).

### 2.3. ETAS^®^ Downregulates Protein Degradation via Ubiquitin-Proteosome System (UPS) and Myostatin in Skeletal Muscle of SAMP8 Mice

**Figure 3:**
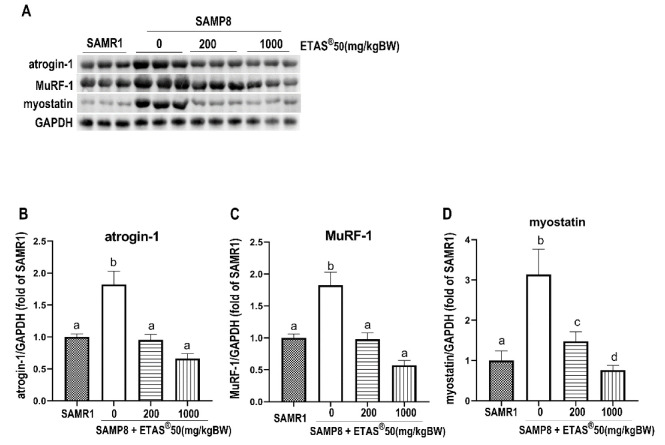
Expression of myopathy-associated proteins. (**A**) Representative images and (**B**–**D**) quantification of atrogin-1, MuRF-1, and myostatin. GAPDH served as an internal control. Results are expressed as means ± SEM, with each group consisting of 4 to 6 mouse samples. Significance (*p* < 0.05) among groups was indicated by different letters.

**Figure 4:**
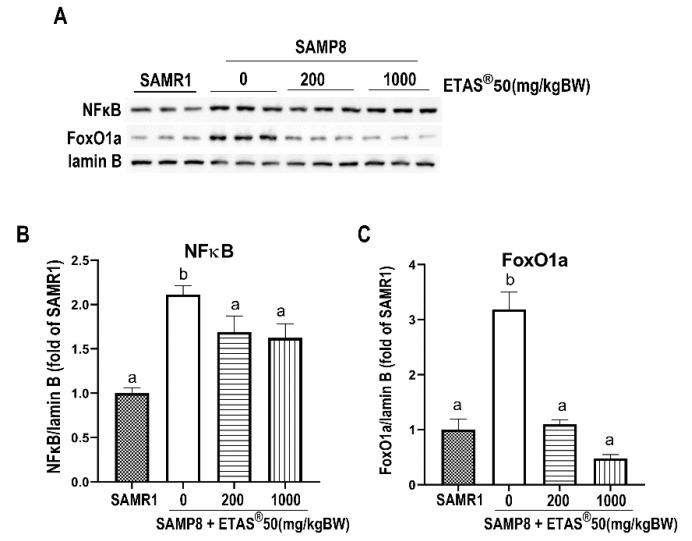
Analysis of nuclear proteins associated with protein degradation. (**A**) Representative images alongside (**B**,**C**) quantification of nuclear NFκB and FoxO1a were presented. Lamin B served as an internal control. Results are presented as mean ± SEM. Each group consisted of 4 to 6 mouse samples. Different letters indicated significant differences (*p* < 0.05) among the groups.

The UPS is a main proteolytic pathway for protein degradation [25]. The excessive expression of the muscle-specific ubiquitin ligases (atrogin-1 and MuRF-1) is believed to contribute to muscle wasting [19,25]. As shown in Figure 3A–C, upregulation of atrophy-related proteins, atrogin-1 and MuRF-1, was observed in non-treated SAMP8 mice compared to age-matched SAMR1 mice (Figure 3A–C). NFκB and forkhead box O1 (FoxO1a) are essential transcription factors that regulate the expression of atrogin-1 and MuRF-1. Notably, their expression levels were significantly elevated in the skeletal muscle of non-treated SAMP8 mice in comparison to age-matched SAMR1 mice (Figure 4A–C). The consistently elevated NFκB- and FoxO1a-mediated atrogin-1 and MuRF-1 were downregulated upon ETAS^®^50 (200 and 1000 mg/kg BW) supplementation in SAMP8 mice (Figure 3A–C and Figure 4A–C). Myostatin, an inhibitory regulator of muscle growth [26], was significantly increased in non-SAMP8 treated mice, and the elevation was significantly limited by the ETAS^®^50 (200 and 1000 mg/kg BW) supplementation (Figure 3D). The inhibited NFκB- and FoxO1a-mediated atrogin-1 and MuRF-1 in conjunction with limited elevation of myostatin contribute to the mitigation of muscle atrophy by ETAS^®^ supplementation.

### 2.4. ETAS^®^ Regulates Mitochondrial Quality Control and Autophagic Flux in Skeletal Muscle of SAMP8 Mice

**Figure 5:**
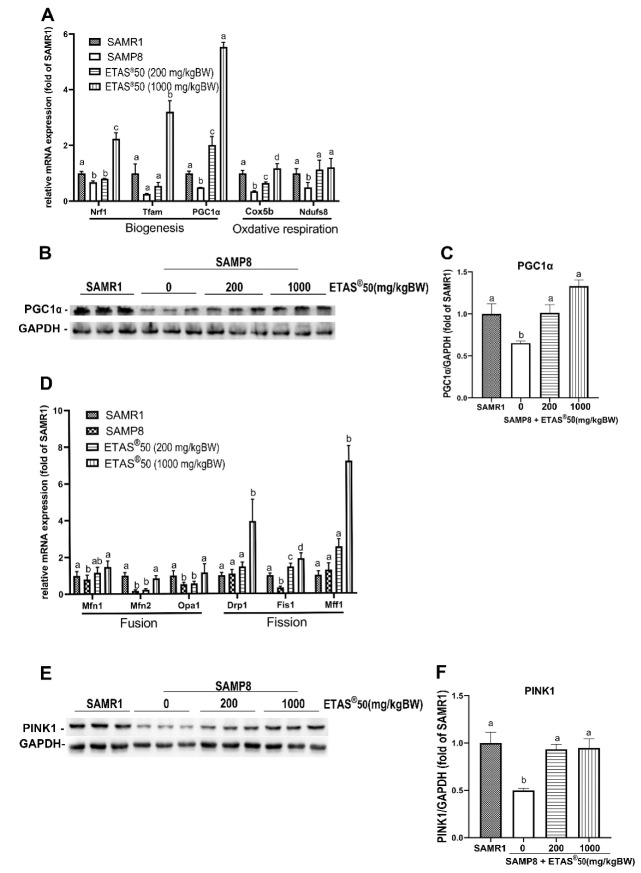
Expression of mitochondrial-associated genes and proteins. (**A**) Quantification of mitochondrial biogenesis (*Nrf1*, *Tfam*, and *PGC1α*) and oxidative respiration (*Cox5b* and *Ndufs8*) mRNA. (**B**) Representative images and (**C**) measurement of PGC1α levels. (**D**) Measurement of mRNA for fusion (*Mfn1*, *Mfn2*, and *Opa1*) and fission (*Drp1*, *Fis1*, and *Mff1*). (**E**) Sample images and (**F**) measurement of PINK1 levels. Each group consisted of 4 to 6 mouse samples. Results were expressed as means ± SEM. Significance (*p* < 0.05) among groups was denoted by different letters.

**Figure 6:**
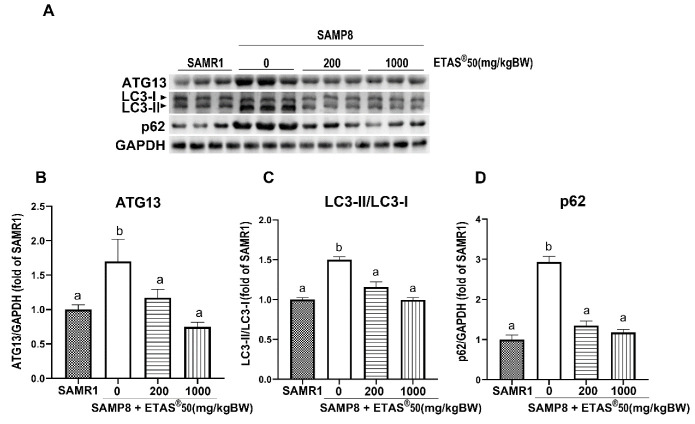
Expression of autophagy-related proteins. (**A**) Representative images and (**B**–**D**) quantification of ATG13, LC3 subunits, and p62. GAPDH served as a reference control. The results were presented as means ± SEM. Each group consisted of 4 to 6 mouse samples. Different letters indicated significance (*p* < 0.05) between groups.

Our previous study has revealed that mitochondrial quality control (biogenesis, dynamics, and redox status) and autophagic flux are disrupted throughout the progression of sarcopenia in SAMP8 [22]. The mRNA (Figure 5A) and protein (Figure 5B,C) for peroxisome proliferator-activated receptor-γ coactivator 1α (PGC1α) were downregulated, along with genes associated with mitochondrial biogenesis [nuclear respiratory factor 1 (*Nrf1*), mitochondrial transcription factor A (*Tfam*)], oxidative respiratory genes [cytochrome c oxidase subunit 5B (*Cox5b*), and NADH: ubiquinone oxidoreductase core subunit S8 (*Ndufs8*)] (Figure 5A) in the skeletal muscle of non-treated SAMP8 mice when compared to age-matched SAMR1 mice. Elevated expression of PGC-1α accompanied by upregulation of Nrf1 and Tfam observed in ETAS^®^-treated groups indicates that ETAS^®^ promotes mitochondrial biogenesis (Figure 5A–C). The levels of mRNA for the mitochondrial fusion genes, mitofusin 1 (*Mfn1*), mitofusin 2 (*Mfn2*), and mitochondrial dynamin-like GTPase (*Opa1*), decreased in the skeletal muscle of non-treated SAMP8 mice (Figure 5D). ETAS® improved the mitochondrial fusion/fission dynamics via regulation of both fusion (*Mfn1*, *Mfn2,* and *Opa1*) and fission genes [fission, mitochondrial 1 (*Fis1*), and mitochondrial fission factor 1 (*Mff1*)] (Figure 5D). Reduced oxidative respiratory genes (*Cox5b* and *Ndufs8*) observed in non-treated SAMP8 mice were restored in the ETAS^®^50 (200 and 1000 mg/kg BW) supplementation mice (Figure 5A). The expression of PTEN-induced kinase 1 (PINK1) protein, which regulates the maintenance of mitochondrial quality, was significantly decreased in non-treated SAMP8 mice. However, this expression was restored to levels observed in SAMR1 mice following treatment with ETAS^®^50 at doses of 200 mg/kg and 1000 mg/kg BW in SAMP8-treated mice (see Figure 5B,E). Taken together, ETAS^®^ maintains the mitochondrial quality control in aging. Autophagy induction was observed in non-treated SAMP8 mice, as indicated by a significant increase in ATG13 and the LC3-II/LC3-I ratio (Figure 6A–C). However, the accumulation of p62 (Figure 6A,D) suggested that autophagic flux was impaired in non-SAMP8 treated mice. The impairment of autophagic flux was restored by ETAS^®^50 (200 and 1000 mg/kg BW) in SAMP8-treated mice through decreased ATG13, LC3-II/LC3-I, and p62 (Figure 6A–D).

## 3. Discussion

This is the first study to demonstrate that ETAS^®^ effectively alleviates sarcopenia by regulating signaling pathways essential for protein turnover and mitochondrial quality in senescence-accelerated mouse prone 8 (SAMP8) mice. The SAMP8, a well-established animal model for studying sarcopenia, exhibits typical features of sarcopenia, indicated by skeletal muscle atrophy, decreased muscle strength, and reduced muscle mass [17,22]. In the present study, supplementation with ETAS^®^ not only significantly increased the gastrocnemius muscle mass but also enhanced grip strength in SAMP8 mice (Figure 1), which is attributed to the positive protein turnover (Figure 2 and Figure 3) and to the improvement of mitochondrial quality (Figure 5 and Figure 6) in aging.

Skeletal muscle atrophy occurs in Akt1/2 double-knockout mice, accompanied by a decrease in mTOR/p70S6K activity [27], highlighting the critical role of this signaling pathway in maintaining muscle mass. mTOR serves as a key regulator of muscle protein synthesis [24]; when activated (by factors such as growth factor stimulation or resistance exercise), it phosphorylates p70S6K, which in turn promotes ribosomal biogenesis, enhances mRNA translation efficiency, and accelerates muscle protein synthesis [24,28]. AKT, a central molecule, controls both protein synthesis via mTOR//p70S6K and protein degradation via FoxO transcription factor [29]. The PI3K/Akt pathway prevents the induction of muscle-specific requisite atrophy mediators, atrogin-1 and MuRF-1, through Akt-mediated inhibition of FoxO1, thereby blocking MuRF-1 and atrogin-1 upregulation [30]. In aging SAMP8 mice, studies have shown that inhibition of AKT activity promotes the nuclear translocation of FoxO, which subsequently upregulates the transcription of muscle atrophy-related genes atrogin-1 and MuRF-1, thereby enhancing protein degradation [22,29]. In the present study, ETAS^®^ supplementation significantly improved grip strength and increased gastrocnemius muscle mass (Figure 1B,C), accompanied with upregulated PI3K/Akt/mTOR/p70S6K activity (Figure 2). It also downregulated the expression of atrogin-1 and MuRF-1 (Figure 3A–C) via inhibiting the elevated nuclear NFκB and FoxO1a in SAMP8 mice (Figure 4), indicating that ETAS^®^ maintains the positive protein balance in aging and mitigates aging-related muscle atrophy.

Myostatin is a well-known inhibitor of muscle growth [26,31], and its suppression leads to enhanced Akt/mTOR signaling and decreased FoxO activity [17,32]. Our study also found that ETAS^®^ suppressed the expression of myostatin (Figure 3A,D), a negative upstream regulator of the Akt/mTOR/p70S6K pathway [31], thereby further promoting muscle protein synthesis. Together, the findings indicate that ETAS^®^ has protective effects against muscle atrophy via simultaneously promoting protein synthesis and inhibiting protein degradation, highlighting its potential as an intervention for combating sarcopenia. Due to the limitations of this study, we did not examine muscle fiber diameter or changes in skeletal muscle fiber type. However, the observed improvements in grip strength, increased muscle mass, and activation of protein metabolism pathways collectively support the potential of ETAS supplementation to alleviate and improve senescence-induced sarcopenia. Additional research is needed to confirm the effects of ETAS on the structure of muscle fibers and the composition of specific muscle fiber types.

Mitochondria play a critical role in skeletal muscle health, as their functional state directly affects muscle energy supply, metabolism, and overall physiological function [20,33]. Aging is associated with progressive mitochondrial dysfunction, which is closely linked to the development of sarcopenia [20]. Our group found that SAMP8 mice showed disrupted mitochondrial quality control in skeletal muscle, which was characterized by a decreased expression of genes related to mitochondrial biogenesis, mitochondrial dynamics, and reduced autophagic flux [22]. Mitochondrial dysfunction leads to insufficient ATP production, negatively affecting muscle contraction, endurance, and overall muscle function [20]. Consistent with these findings, the current study demonstrates that ETAS^®^ supplementation effectively restores mitochondrial quality control, indicated by enhancement of mitochondria biogenesis genes, oxidative respiration genes, mitochondrial dynamics genes, PINK1 protein (Figure 5), and improvement of autophagic flux (Figure 6). ETAS® significantly upregulated the mRNA expression of *Pgc1α*, *Nrf1*, and *Tfam*, key regulators of mitochondrial biogenesis, suggesting an improvement in mitochondrial generation and maintenance. Our results demonstrated that ETAS^®^ inhibits NFκB and myostatin while promoting PGC-1α; these findings align well with the current literature [34] that highlights the interplay among these molecules in muscle metabolism and inflammation. Furthermore, ETAS^®^ restored the mRNA expression of *Cox5b* and *Ndufs8*, critical components of the electron transport chain, which may contribute to enhanced oxidative phosphorylation and ATP production. In addition to biogenesis, mitochondrial dynamics were also favorably modulated by ETAS^®^ (Figure 5). The mRNA expression of *Mfn1*, *Mfn2*, and Opa1 was upregulated, suggesting enhanced mitochondrial fusion, which is essential for maintaining mitochondrial integrity and function. Moreover, ETAS^®^ influenced fission regulation through modulation of *Fis1* and *Mff1* mRNA expression, further supporting its role in maintaining mitochondrial network stability. Another key finding is the restoration of the PINK1 protein, a central regulator of mitophagy [35], in SAMP8 mice by ETAS^®^, suggesting that ETAS^®^ facilitates the selective clearance of damaged mitochondria, promoting mitochondrial turnover, and reducing cellular stress (Figure 5B,E). Our previous study indicated that the deficient fusion between autophagosomes and lysosomes was evidenced in SAMP8 mice [22,36]. In the current study, the induction of autophagy indicated by elevated levels of ATG13 and LC3-II and impairment of autophagic flux indicated by increased P62 were observed in SAMP8 (Figure 6). ETAS^®^ supplementation improved autophagic flux, as evidenced by balanced ATG13, LC3, and p62 (Figure 6), indicating efficient lysosomal degradation and recycling of dysfunctional mitochondria. Taken together, our findings demonstrate that ETAS^®^ enhances mitochondrial quality control by promoting mitochondrial biogenesis, regulating mitochondrial dynamics, and facilitating mitophagy, ultimately supporting mitochondrial function in aging skeletal muscle. These improvements may help counteract aging-related mitochondrial dysfunction that contributes to muscle decline.

ETAS^®^ has been reported to exert anti-inflammatory effects by inhibiting NFκB [9,10] and pro-inflammatory cytokines [3,10,11] and displays antioxidant properties by upregulating HSP70 [2,6,12] and maintaining redox balance [2,12]. Increased lipid accumulation in muscle tissues is suggested as a potential inflammatory state in skeletal muscle [37]. NFκB, a key regulator of inflammation, plays a pivotal role in skeletal muscle metabolism by modulating the ubiquitin-proteasome system, inflammatory responses, and myogenesis [38]. Activation of NFκB leads to the transcription of pro-inflammatory cytokines contributing to chronic low-grade inflammation and muscle catabolism, which are characteristic features of sarcopenia [38]. Studies have shown that ETAS^®^ exerts anti-inflammatory effects in various models. In UVB-irradiated human dermal fibroblasts, ETAS^®^ suppresses NFκB nuclear translocation, further supporting its anti-inflammatory properties [11]. In the present study, we found that supplementation of ETAS^®^ attenuated NFκB nuclear translocation in the skeletal muscle of SAMP8 mice (Figure 4), indicating that ETAS^®^ may mitigate aging-related muscle atrophy by suppressing NFκB-mediated inflammation. Given that NFκB activation is closely linked to the upregulation of inflammatory cytokines and muscle degradation pathways, our findings suggest that ETAS^®^ may exert protective effects against sarcopenia by modulating NFκB signaling and reducing inflammation-associated muscle loss. In addition to its role in suppressing catabolic signaling, ETAS^®^ is also well-known for its ability to induce the expression of HSP70 [2,6,12,39], a stress-inducible chaperone protein that facilitates protein folding, stability, and turnover [40,41]. HSP70 is essential for maintaining skeletal muscle integrity and function, as it promotes protein synthesis and protects muscle cells from damage [40]. Previous research indicates that HSP70 expression enhances myotube formation and protects C2C12 myoblasts from cellular stress, further supporting its crucial role in muscle maintenance and repair [42].

It has been demonstrated that 3-Alkyldiketopiperazines, known as asparaprolines—specifically cyclo (L-Phe-L-Pro), cyclo (L-Tyr-L-Pro), and cyclo (L-Leu-L-Pro), along with 5-hydroxymethyl-2-furfural (5-HMF) and its potent derivative, asfural, are key components present in ETAS^®^ extracts. These compounds highlight the unique and beneficial qualities of ETAS^®^ and underscore its potential impact in various applications. HMF is a well-known byproduct of the Maillard reaction. Recent studies have shown that 5-HMF possesses multiple biological activities, including antioxidant, anti-sickling, and anti-inflammatory properties. Ito et al. (2013) and Inoue et al. (2020) reported that ETAS^®^, HMF, asfural, as well as asparaprolines all presented a good induction activity on HSP70 mRNA in HL-60 cells [39,43]. Ciarlone et al. (2023) demonstrated that 5-HMF improves skeletal muscle force production under hypobaric hypoxia conditions through inhibition of superoxide production [44]. In addition to HMF, asfural, and asparaprolines, asparagus extracts have been reported to contain polyphenolic compounds such as quercetin [1]. Notably, Cui et al. (2022) reported that quercetin improves mitochondrial quality and mitigates aging-related decline, suggesting that asparagus-derived quercetin may contribute to mitochondrial protection [45]. Consistently, Ho et al. (2023) demonstrated that ETAS^®^ supplementation increased mitochondrial activity, and further evidence has shown that ETAS^®^ treatment enhances mitochondrial function in bovine granulosa cells [2]. These findings collectively support the hypothesis that ETAS^®^ may exert beneficial effects on mitochondrial quality control through multiple bioactive compounds, although the specific constituents responsible remain to be fully elucidated. Given its antioxidant and anti-inflammatory properties, ETAS^®^ may contribute to alleviating aging-related sarcopenia by protecting skeletal muscle from proteolytic degradation, enhancing protein synthesis, promoting mitochondrial quality, and improving overall muscle functionality.

## 4. Materials and Methods

### 4.1. Animals and Experimental Design

The 12-week-old SAMR1 and SAMP8 mice were housed in the animal facility of the Department of Nutrition at Providence University under conditions approved by the Institutional Animal Care and Use Committee (20180605-A002). Lighting was controlled with an automatic timer, providing a 12-h light and dark cycle. The animal room was maintained at 22 ± 2 °C and a relative humidity of 60–70%.

SAMR1 mice were assigned as a control group and fed a standard chow diet (AIN-93M, Bio-Serv Co., Flemington, NJ, USA) for 12 weeks. The SAMP8 mice were divided into groups receiving a control diet (*n* = 8) or diets supplemented with ETAS^®^50 at 200 mg/kg body weight (BW) (*n* = 8) and 1000 mg/kg BW (*n* = 8) daily for 12 weeks. ETAS^®^50, a spray-dried powder containing 50% [asparagus extract–solid content] and 50% dextrin (as a filler), was kindly provided by the Amino Up Co., Ltd. (Sapporo, Hokkaido, Japan). The extraction process of ETAS^®^ was conducted according to the methodologies in the studies by Ito et al. (2013, 2014) [12,43]. The manufacturing of ETAS^®^ adheres to Good Manufacturing Practice (GMP) standards for dietary supplements and complies with ISO 9001:2015 [46] and ISO 22000:2018 [47] quality and food safety management systems. As a result, the ETAS^®^ material used in this study was sourced from the same commercial batch as that used in other published research, ensuring consistency in composition and quality. All groups were provided with free access to food and water. Food intake and body weight were recorded weekly. At 24 weeks of age, the mice were euthanized via carbon dioxide anesthesia, followed by decapitation. Gastrocnemius muscle tissues were collected and stored in a liquid nitrogen container for further analysis.

### 4.2. Grip Strength Analysis

A Grip Strength Meter (model #BIO-GS3, BIOSEB Company, Oakmont, PA, USA) was utilized to assess the forelimb grip strength of the mice undergoing various treatments. Each mouse was placed on a grid and gently pulled by its tail with increasing force until it could no longer maintain its grip on the grid. The highest grip strength achieved by the mouse was recorded, with the muscle grip strength defined as the maximum weight (in grams) shown on the device. The forelimb grip strength assessment was carried out when the mice reached 24 weeks of age. Three trials were conducted for each condition, and the results were averaged.

### 4.3. Tissue Homogenization, Nuclear Protein Extraction, and Western Blot Analysis

Fifty milligrams of the gastrocnemius muscle were combined with 500 µL of lysis buffer consisting of 0.1% Triton X-100, 50 mM 4-(2-hydroxyethyl)-1-piperazineethane sulfonic acid (HEPES), 150 mM sodium chloride, and 10 mM ethylenediaminetetraacetic acid. This solution also contained a protease inhibitor from Roche Company (Basel, Switzerland), along with 1 mM sodium orthovanadate, 30 mM sodium fluoride, and 10 mg/mL phenylmethanesulfonylfluoride. Minced muscle tissues were homogenized seven times, each time continuing for 15 s and resting for 10 s. Put The homogenized tissue was put on ice and shaken for 30 min, followed by centrifugation at 14,000 rpm for 15 min. The supernatants were collected and stored at −80 °C until use.

To extract nuclear proteins (NFkB and FoxO1a), 50 mg of gastrocnemius muscle was chopped and homogenized in 250 µL of pre-extraction buffer (#ab113474, Abcam Plc., Cambridge, MA, USA). The total lysates were then centrifuged at 4 °C at a speed of 12,000 rpm for 10 min. After discarding the supernatant, the remaining pellets were mixed with the extraction buffer and sonicated for three 10-s intervals. After centrifugation of the lysates, the supernatants were collected at 14,000 rpm for 10 min.

Appropriate protein amounts (30–60 μg) were subjected to sodium dodecyl sulfate-polyacrylamide gel electrophoresis. After electrophoresis, proteins were transferred to a polyvinylidene difluoride membrane. The membranes were incubated in a blocking buffer (5% non-fat milk, 20 mM Tris, pH 7.6, 150 mM NaCl, 0.1% Tween-20) for one hour at room temperature, followed by overnight incubation with the primary antibody (Table 1) at 4 °C. The blots were rinsed twice every five minutes using tris-buffered saline with 0.1% Tween 20 and were subsequently treated with a secondary antibody for 60 min. Immuno-detection utilized the ECL detection kit (Amersham Plc., Little Chalfont, Buckinghamshire, UK) for HRP-conjugated secondary antibodies. Lamin B or Glyceraldehyde 3-phosphate dehydrogenase (GAPDH) was used as a loading control.

### 4.4. Real-Time Polymerase Chain Reaction (PCR)

Total RNA was extracted from 50 mg of gastrocnemius muscle using TrizolTM reagent (InvitrogenTM, Thermo Fisher Scientific Inc., Waltham, MA, USA), as per the manufacturer’s guidelines. An iScriptTM cDNA synthesis kit (Bio-Rad Laboratories Inc., Hercules, CA, USA) was utilized to produce complementary DNA (cDNA). The cDNA mixtures were subsequently amplified with the FastStart SYBR Green Master enzyme (Thermo Fisher Scientific) to analyze the relative expression of each RNA through real-time PCR using a StepOnePlusTM machine (ABI system, ThermoFisher Scientific Inc., Waltham, MA, USA). The real-time PCR protocol was executed according to the manufacturer’s specifications. The primer sequences used in the reactions are listed in Table 2.

### 4.5. Statistical Analysis

The protein and mRNA expression data are presented as fold changes relative to the SAMR1 group, which is set as 1 for comparison. The results are presented as the mean ± standard error of the mean (SEM) and analyzed by IBM SPSS Statistics 20 software. One-way ANOVA was analyzed for a statistical difference and corrected by the LSD test [48]. *p* < 0.05 indicates a statistical difference.

## 5. Conclusions

Our study demonstrates that long-term ETAS^®^ supplementation effectively mitigates aging-related muscle loss and strength decline. These effects are achieved through the positive protein turnover via upregulation of PI3K/Akt/mTOR signaling and downregulation of FoxO- and NF-κB-mediated atrogin-1 and MuRF-1 expression, and the improvement of mitochondrial quality and autophagic flux. These findings suggest that ETAS^®^ holds promise as a potential intervention for age-associated sarcopenia and muscle atrophy.

## Figures and Tables

**Table 1:** Primary antibody list.

Antibody	Dilution	Brand
PI3K (p110a, #4249), p-Akt (Thr308, #4056), Akt (#9272), p-mTOR (Ser2448, #5536), mTOR (#2983), p-p70S6K (Thr389, #9205), p70S6K (#2708), FoxO1a (#2880)	1:1000	Cell Signaling Technology (Danvers, MA, USA)
GAPDH (GTX100118), NFκB (GTX102090), MuRF-1 (GTX110475), and p62 (GTX100685)	1:2000 1:1000	GeneTex Inc. (Irvine, CA, USA)
Atrogin-1 (ab168372), Myostatin (ab203076), and lamin B (ab16048)	1:1000	Abcam (Cambridge, MA, USA)
PGC1α (sc-13067)	1:1000	Santa Cruz Biotechnology Inc. (Santa Cruz, CA, USA)
LC3B (14600-1-AP), ATG13 (18258-1-AP)	1:1000	proteintech ^®^ (Proteintech Group Inc., Rosemont, IL, USA)
PINK1 (A7131) β-actin (AC006)	1:1000 1:10,000	Abclonal^®^, BioAb Co., Ltd., New Taipei City, Taiwan

**Table 2:** Real-time PCR primers list.

Gene	Forward Primer (5′–3′)	Reverse Primer (5′–3′)
Mfn1	AGTCAGCGGTGAAAGCAAAGT	GGTCTTCCCTCTCTTCCATTGAAT
Mfn2	ATATAGAGGAAGGTCTGGGCCG	CCGCATAGATACAGGAAGAAGGG
OPA1	TGACAAACTTAAGGAGGCTGTG	CATTGTGCTGAATAACCCTCAA
Drp1	CGGTTCCCTAAACTTCACGA	GCACCATTTCATTTGTCACG
Fis1	AGCTGGTTCTGTGTCCAAG	TGTTCCTCTTTGCTCCCTTTG
Mff1	CTAATCTTTCCTCTGCCCGT	GATGAGGATTAGAAGTGGCGG
PGC1α	ACTATGAATCAAGCCACTACAGAC	TTCATCCCTCTTGAGCCTTTCG
Nrf1	ACAGATAGTCCTGTCTGGG	TGGTACATGCTCACAGGGA
Tfam	AAGACCTCGTTCAGCATAT	TTTTCCAAGCCTCATTTACAAGC
COX5b	TTCAAGGTTACTTCGCGGAGT	CGGGACTAGATAGGGTCTTCC
Ndufs8	AGTGGCGGCAACGTACAAG	TCGAAAGAGGTAACTTAGGGTCA
GAPDH	AGGTCGGTGTGAACGGATTTG	TGTAGACCATGTAGTTGAGGTCA

## Data Availability

The original contributions presented in this study are included in the article. Further inquiries can be directed to the corresponding author.

## Abbreviations

ATG13, autophagy-related protein 13; Cox5b, cytochrome c oxidase subunit 5B; ETAS®, A standardized extract of *Asparagus officinalis* stem; Fis1, fission, mitochondrial 1; FoxO, forkhead box O; LC3, microtubule-associated proteins 1A/1B light chain 3B; MAFbx/atrogin-1, muscle atrophy F-box (MAFbx)/atrogin-1; mTOR, mammalian target of rapamycin; Mff1, mitochondrial fission factor 1; Mfn1, mitofusin 1; Mfn2, mitofusin 2; MuRF-1, Muscle RING-finger protein-1; NFκB, nuclear factor kappa-B; Ndufs8, NADH: ubiquinone oxidoreductase core subunit S8; Nrf1, nuclear respiratory factor 1; SAM, senescence-accelerated mice; Tfam, mitochondrial transcription factor A; Opa1, OPA1 mitochondrial dynamin like GTPase; PINK1, PTEN induced kinase 1; p70S6K, The 70-kDa ribosomal protein S6 kinase; PGC1α, peroxisome proliferator-activated receptor-α coactivator 1α.
